# Caring for Dying and Meeting Death: Experiences of Iranian and Swedish Nurses

**DOI:** 10.4103/0973-1075.68405

**Published:** 2010

**Authors:** Sedigheh Iranmanesh, Karin Axelsson, Stefan Sävenstedt, Terttu Häggström

**Affiliations:** Razi Faculty of Nursing and Midwifery, Kerman Medical University, Kerman, Iran; 1 Department of Health Science, Luleå University of Technology, Luleå, Sweden

**Keywords:** Caring for dying, Iranian nurses, Oncology, Palliative care, Swedish nurses

## Abstract

**Objective:**

Our world is rapidly becoming a global community, which creates a need to further understand the universal phenomena of death and professional caring for dying persons. This study thus was conducted to describe the meaning of nurses’ experiences of caring for dying people in the cultural contexts of Iran and Sweden.

**Materials and Methods:**

Using a phenomenological approach, phenomenon of caring for dying people was studied. Eight registered nurses who were working in oncology units in Tehran, Iran and eight registered nurses working in hospital and home care in North part of Sweden were interviewed. The interviews were analyzed using the principles of phenomenological hermeneutics.

**Results:**

The findings were formulated based on two themes included: (1) “Sharing space and time to be lost”, and (2) “Caring is a learning process.

**Conclusions:**

The results showed that being with dying people raise an ethical demand that calls for personal and professional response, regardless of sex, culture or context. The physical and organizational context must be supportive and enable nurses to stand up to the demands of close relationships. Specific units and teamwork across various personnel seem to be a solution that is missing in Iran.

## INTRODUCTION

Human lives include a steady existential questioning and search for meaning in which death is an inevitable and natural phenomenon. A life threatening disease such as cancer involves patients and their families, thus the family members who have a supportive role and often assume caring work become exhausted. Even if people nowadays often prefer to die at home and to be cared for by their family members, they still need professional services and supports.[1] This need has been approached by establishing palliative care since 1960s. Palliative care affirms life and considers dying as a normal process, intends neither to hasten nor postpone death, offers a support system to help patients live as actively as possible until death, uses a team approach to address the needs of patients and their families, and offers a support system to help the family cope during the patients’ illness and in their own bereavement.[2]

According to Roach[3] caring is the human way of being and Leininger[4] claims that caring is the essence of nursing, even if different and common meanings and expressions of care vary culturally. The nurses’ understanding of care, their attitudes and behaviors become more important in the context of palliative care where a variety of feelings and experiences are evoked in the face of death. Different views on death are based on the differences in various cultures. The prospect of mortality could be both as the foundation on which culture is built[5] and the primary challenge to the search for meaning.[6]

Trans-culture is a form of culture created not from within separate spheres, but in the holistic forms of diverse cultures. It is based on the principle that a single culture, in and of itself, is incomplete and requires interaction and dialogue with other cultures.[7] Thus, the understanding of care from a single nurse’s point of view and its content depends on the life story of that nurse.[8] Anyhow, our world is rapidly becoming a global community, which creates a need to further understand the universal phenomena of death and professional caring for dying persons. It is also essential to know more about prevailing views on death and dying among future and presently working registered nurses in different cultures. Reviewing literature revealed a lack of trans-cultural palliative care studies that show how registered nurses experience caring for dying people and their relatives within different cultural contexts. Only one study was found on the topic of trans-cultural care for dying persons that was conducted in Greece and Hong Kong.[9] This study thus was conducted to describe the meaning of nurses’ experiences of caring for dying people in the cultural contexts of Iran and Sweden.

### Contexts of the study

The sample of nurses in this study comes from two different cultural contexts. Sweden is a part of European culture, whereas Iran is a part of Middle Eastern culture. The respective cultures in Iran and Sweden differ with regard to the dominating religion and also with regard to how influential the religion is in the everyday life of the people. Many religions are represented in both countries, but the majority of the people in Iran follow Islam, and the majority in Sweden follow Christianity.

Cheraghi, Payne and Salsali[10] point out that there are no hospice-care units in Iran like those in Western countries. Palliative care, including hospice care, is well established in Sweden. Universities provide special training courses in palliative nursing and support the quality and development of palliative care through teaching and research.[11] There is a difference in the inclusion of the subjects about death and palliative care in the national curriculum between the two countries. In Iran, the overall national curriculum for registered nursing education includes 2-4 h of theoretical education about death and care for a dead body. They had no special education or training in the care for dying persons, although the topic was included in other courses. Swedish nursing students have about 40 h education and reflective supervision during their practical education.

## MATERIALS AND METHODS

Using a phenomenological approach, phenomenon of caring for dying people was studied from the viewpoint of the individual. The methodology seeks to focus on a person’s experience. It is based on that person’s previous understanding and knowledge, which are embedded in culture and history. Husserl[12] described phenomenology as a turn into the things themselves. It was described as a view of the world based on experience. The overall aim of life-world research, according to Dahlberg *et al*.,[13] is to describe and elucidate the lived world in a way that expands our understanding of human being and human experience. In this sense, phenomenology and hermeneutics are each other’s prerequisites.[14] Phenomenological hermeneutics helps us to develop the critical understanding of a studied discourse. In other words, it helps us to obtain knowledge of the essential meaning of a lived experience.[15]

Eight registered nurses who were working in oncology units in Tehran, Iran and eight registered nurses working in hospital and home care in North part of Sweden were interviewed. The Iranian interviewees’ ages were between 25 and 50 years. Four of them were men. All of them were registered nurses with BSc degrees in nursing. Their mean oncology experience was 7 years. The ages of Swedish interviewees ranged from 35 to 58 years. They had a mean of 12 years of experience working with palliative care.

In the qualitative studies, open, structured interviews were conducted with a narrative methodology.[16] The participants were asked to narrate their experience of caring for dying people. Clarifying and encouraging questions were used, such as “Please, explain more about...”? or “Can you give an example”? The interviews were conducted in the informants’ mother tongue and by the members of target language (S.I in Iran and T. H., and S. S in Sweden). During the interviews, the researchers tried to strike a balance between listening to the stories told by the participants and keeping the focus of the stories on the aim. The interviews lasted between 45 and 60 min.

One ethical consideration was the assurance of confidentiality for the participants. These participants were informed about the purpose of the study and assured that their participation was voluntary. Terminally ill persons and the experiences related to them are an emotionally charged topic and may be a painful reminder of previous experiences. This risk was handled by the researchers’ attentive and sensitive attitude toward the interviewees’ emotional reactions. The researchers also gave the participants sufficient time to consider their participation in the study.

## RESULTS

The interviews were analyzed using the principles of phenomenological hermeneutics. From the transcribed data, a selective reading approach was adopted, meaning that we read the text several times and asked: ‘What statements or phrases seem particularly essential or revealing about the phenomenon or experience described? These meaning units were underlined and highlighted. These meaning units were thereafter condensed and translated into English. The findings were formulated based on reflections on the essential themes that characterized the phenomenon. The hermeneutic process involved a systematic analysis of the whole text, systematic analysis of parts of the text, and comparison of the two interpretations for conflicts and an understanding of the whole in relationship to its parts.[17] Interpretation of the written narratives moved back and forth between the whole and parts of the texts. The goal was to find commonalities in meanings, situations and lived experiences. Categories that clearly describe the themes were identified.

### Sharing space and time to be lost

According to the texts, professional care for people at the end of their lives means sharing space and time with the people who are dying and their relatives, which all occurs within a limited time. Nurses experienced that patients and their families were units that should not be separated and cared for in parts. Nurses share the space and time with dying persons by creating a close relationship and meeting their spritual needs.

### Creating a close relationship

The way nurses choose to share the space and time with persons and families was through taking part in patient’s daily life matters and getting close to the family as a whole. Iranian nurses expressed that caring for dying people required them to have a mutual close relationship with the patients and their relatives. Swedish nurses also described caring for dying people as meeting patients as unique persons through creating a trustful and close relationship. Since the patient and his or her family members were seen as united, the nurses’ relationships included the family. The relationship with dying persons and their family members in both groups involved addressing the family members’ personal needs and making efforts to meet their concerns with counselling and support.

In palliative care time is limited, so we have to use the time to get close to the patients and their relatives in order to meet the whole patients.

Spending time with dying people, listening to their concerns, using touch, and talking with them supported the nurses’ interactions with the persons and even went so far as to have a healing effect. Nurses also talked with the family about care at the end of life. Nurses’ physical care often was combined with emotional care. Swedish nurses listened to the patients and became aware of their wishes. They also comforted the dying persons. Overall, this approach constituted a means to become aware of a person’s needs and preferences when facing death. It also allowed nurses to become aware of what the people perceived through their senses to be tasteful and beautiful.

I facilitated her leaving, she went to her village and died after three days, the feeling of being able to understand her last will was really satisfying.

### Meeting spiritual needs

In both countries, meeting the spiritual needs of dying people was seen as an important part of nurses’ daily care. It meant searching for the meaning together with dying persons and their families. This is the best way to assist them in coping with the difficult situation they faced. Sharing discussion about existential matters, values and beliefs served as a means to alleviate dying persons’ spiritual pains and anxieties. When nurses in both countries became involved in such matters, they attempted to respect the dying persons’ beliefs and faiths even if their own views were different. They felt that patients and family members were all involved in their own search for meaning.

I listened to the patient when he talked about his life. He believed that cancer is the result of his work and his bad behavior toward others.

Nurses were also involved in the patients’ and their family members’ search for the meaning of death in their present lives. Iranian nurses’ religious beliefs seemed to give them insight in order to provide spiritual care to the patients and their families. Values and religious beliefs as well as practices gave new meaning to the nurses’ professional life. Their spiritual foundation and religious beliefs assisted them in a positive way to cope with and provide meaning to the work at the oncology unit. They also referred to specific religious beliefs that guided their practice or their approach to the patients. They talked about their beliefs on a life after death, a circular process of life, death as a divine order, and that such a belief facilitates their caring relationship with people at the end of their lives.

We are Muslim and believe in life after death. Referring to the religious beliefs is the best way to support a dying person in order to experience an easy death.

Among Swedish participants, many of them emphasized that they have no religious faith, but they expressed deep appreciation of the comforting role of religious ministers and spiritual comfort for patients. They stated that getting into the conversation about existential matters among persons who have different values needs to be honest and make a mutual trust; otherwise this type of conversation will never take place. While some nurses expressed their individual experiences of getting involved in conversation about existential matters, the other stated that if patients were interested to get into such a conversation, they left the issues to the priest.

I suggested them to go to hospital church; it was very good for them, because they did not meet just a priest, but something that he really needed.

### Caring is a learning process

One meaning of Iranian and Swedish nurses’ experiences of caring for dying people was that caring was considered to be a learning process. Caring as a learning process meant developing caring as well as self consciousness.

### Developing caring consciousness

Nurses in both countries stated that caring for terminally ill persons required them to develop their own personal ways of caring. They experienced that working with patients in the oncology wards required them to be closer and more compassionate to them compared to care giving work in the other wards. Iranian nursesexpressed that theylearned to be compassionate in a process that took several years.

I learned from people that they need us to be there and spend time with them. Over time I realized that this is the most important thing in the care.

Swedish nurses expanded consciousness through an ongoing learning process, as expressed by meeting each person as a unique being. They stated that in palliative care context they are “persons” whereas in the hospital ward they are one of the “sisters”; one in the flock among the others with white coat and name tag. When they compared, they found the present caring context very stimulating and challenging as it provided a different and better foundation for a person-to-person relationship.

In the hospital you can always behave in the same way, but here you have to adjust and you can not behave in the same way among different persons.

Swedish nurses expressed that caring for dying people require them to work in a team. They stressed the importance of trusting each other and sharing common values among the members of the team about the meaning of patient centred care. Trustful relationships and having shared commonalities constituted a positive climate of care once it could be achieved. Such sharing of values referred to the colleagues and to other professionals involved in the total care of the person who was dying. Working in teams enabled nurses to manage difficult situations and to make decision and it was important to have consensus when important decisions were to be made.

I felt it as a progress in my own way that I could trust on the other members of the team to take care of motherless children and not face a break down myself.

In contrast to Swedish nurses, the Iranian nurses worked individually. They lacked support from other healthcare professionals, so they had to manage all of the patient’s needs by themselves, even the patient’s economic problems.

His economical situation was bad. We collected money for him to pay for thickets and his travel budget.

Iranian nurses felt frustrated because of the lack of palliative care units to focus on people’s special needs during the process of death and dying. Iranian nurses described that palliative care is providing a good quality of life with respect to the dying process and death, while using life saving strategies in an oncology unit is actually prolonging life with no quality:

Actually we just try to extend their life with chemical drugs, even if we see how they suffer from those drugs and how many difficulties they have.

### Developing self consciousness

The learning process of caring was also interpreted by nurses as expanding self-consciousness. Nurses in both Iran and Sweden experienced relations with dying persons that made them change the way they looked at their own lives. They discovered that they had changed their views on many things. Witnessing people’s suffering made them re-examine their own attitudes toward life and accept that incurable disease and death are parts of life. They expressed this as being rewarding, gaining an inner strength, and being more patient with their own personal problems:

I believe that life is not just pleasure; it is also labouring and anxiety provoking. This insight helps me to continue with this work.”

Caring for patients who will soon die and not being able to help them bring feelings of frustration. The nurses had become aware about the fact that there were limitations in what the health care system could provide in order to save a person’s life and that was a discouraging awareness. There was sadness, especially in situations when the nurses identified themselves with the patients’ situation as when the patient was about the same age or had children about the same age as the nurses’ children. Swedish nurses experienced becoming confident and self-reliant with few uncertainties about their ability to care. They learned to keep a balance between being professionals and being close to the people who were dying and their families. Therefore, they were able to handle the anxieties caused by work.

We learn how to control our feelings, trying to be a professional and not to be influenced by anxieties in order to be able to work.

## DISCUSSION

Caring for a dying person is specific in that a dying person’s lifetime is running out. The space that her or his body is occupying will soon be left empty. The alleviation of existential pain requires nurses to be closed and compassionate and view patients as unique people. Facing death or an incurable disease means living with existential pains, feelings of meaninglessness, loneliness, reduced self-respect, and loss of the sense of control.[18] Being a professional, nursing care in the last waiting room is a unique experience. It is different from most of the nursing care in the other wards. Rasmussen[19] suggests that being a hospice nurse has more to do with being than with doing. It is difficult to distinguish between the nurse as a person and a professional.

The existential context demanded nurses to create a close and trusting relationship with dying person and the whole family. Sand and Strang[20] emphasize that respect, empathy and provisional care with mutual togetherness and belonging may decrease the perception of existential loneliness for dying persons and their families. Nurses used touch in situations characterised by anxiety and physical pain. This use could have the greatest impact on bodily discomfort, especially in severe illness. It may reduce bodily suffering since symptoms affect both the mind and soul.[20]

Nurses’ close relationships with dying people in both Iran and Sweden were ethical, unconditional and compassionate. Love and honesty are examples of morality, a common virtue among all nurses in the world.[21] The existential context of caring for dying people demands that nurses have a holistic view of care, including spiritual care. This corresponds with Piles[22] and Carroll,[23] who stated that there is a connection between spiritual care and holistic philosophy. The nurses experienced relieving persons’ anxieties and supported them in their search for meaning. Spirituality is a personal search for meaning and purpose in life. Nurses inspire and motivate individuals to achieve their optimal being.[24] Swedish nurses expressed spirituality as a search for existential meaning. Iranian nurses interchangeably connected spiritual care and religiosity. Swedish nurses’ concerns about patients’ spiritual and existential needs were seen as a part of holistic care. This finding reflects the results of Strang *et al*.[25] and Lundmark.[26] They conclude that holistic care is desirable in Swedish health care.

Nurses viewed caring for dying persons as a worthwhile learning experience. Caring as a learning process among Iranian and Swedish nurses meant developing consciousness. An asymmetry in caring relationships could potentially be unethical if it is not balanced with reciprocity.[27] Personal and professional development can be the main strategy that nurses use to cope with the challenging work that they do. Wengström and Ekedahl[28] claimed that professional development is a coping process that nurses use when caring for persons with cancer.

Nurses in both countries viewed caring for dying persons as an opportunity to re-examine their values about life and death. According to Byrne and McMurray,[29] nurses who work with dying people develop a personal philosophy of death and dying. They also develop a realistic perspective of death and dying. Nurses’ personal development can be viewed as a prerequisite for professional development. In hospice care, the personal dimension of professional identity is important. Personal identity should be considered as a prerequisite for the development of a professional identity.[30]

Most Swedish nurses mentioned their philosophical foundations. These foundations facilitated their caring relationships with dying persons and their family members. Their colleagues in Iran considered religion and religiosity as factors that supported their caring relations with patients and their family members. This reflects the finding of Iranmanesh’ *et al*. study.[31] This study showed that Iranian nurses considered themselves religious and they tended to view death from a more religious perspective. This agrees with Tomás-Sábado and Gόmez-Benito’s[32] statement that what happens after death often is linked to religious issues.

Swedish nurses had high self-esteem and were able to balance closeness and distance. One possible explanation for Swedish nurses’ self-esteem could be that they worked in teams, not individually. Blomberg and Sahlberg-Blom[33] explain that team work is complementing, helping and strengthening one another in a support for facilitating closeness and its balance with distance. Sengin[34] reports that the relationships with co-workers and supervisors in team work, as well as collaboration with physicians in decision- contribute to the nurses’ job satisfaction.

In contrast to Swedish nurses, the Iranian nurses worked individually. There are some possibilities that may have caused the lack of professional or inter-professional team work in the Iranian health care system. Headrick, Wilcock, and Batladen[35] list a number of barriers to inter-professional collaboration such as: differences in schedules and professional routines, differences in requirements, regulations and norms of professional education, fears of diluted professional identity, differences in accountability, payment and rewards as well as concerns regarding clinical responsibility.

Iranian nurses felt frustrated because of the lack of palliative care unit. They experienced the necessity of a patient-centred care unit in order to focus on the people’s quality of life at the end rather than on the life-prolonging technologies. This echoes the other findings that “the opportunities to relieve suffering and help persons achieve meaningfulness from life-limiting conditions are neglected”[36] because of the dominance of technology and treatment concerns in the clinical healthcare system dealing with dying persons.[37]

## CONCLUSION

Professional caregivers must be sensitive and pay attention to the preferences of each unique person’s perceptions through her or his senses. This includes views, tastes, sounds, smells and bodily contact. The ability of a dying person to see a sunset may seem petty, but is important in providing good care for people at the end of their lives. The same goes for the other senses. These circumstances deserve attention in all educational programs and especially in programs dealing with end of life care. To implement holistic care, caregivers must pay attention to patients’ spiritual needs.

The results showed that being with dying people raise an ethical demand that calls for personal and professional response, regardless of sex, culture or context. The physical and organizational context must be supportive and enable nurses to stand up to the demands of close relationships. Specific units and teamwork across various personnel seem to be a solution that is missing in Iran.
